# Evaluation of proper prescribing of cardiac medications at hospital discharge for patients with acute coronary syndromes (ACS) in two Lebanese hospitals

**DOI:** 10.1186/2193-1801-3-159

**Published:** 2014-03-25

**Authors:** Marwan Sheikh-Taha, Zeinab Hijazi

**Affiliations:** Clinical Associate Professor Lebanese American University, P.O. Box: 36, Byblos, Lebanon

**Keywords:** ACS, Cardiac medications, Secondary prevention, Coronary artery disease, CAD

## Abstract

**Background:**

Coronary artery disease (CAD) is the major leading cause of death worldwide. The national practice guidelines from the American College of Cardiology (ACC) and American Heart Association (AHA) promote the use of several medical therapies for secondary prevention for patients with CAD. The purpose of this study was to evaluate whether ACS patients, admitted into two tertiary referral medical centers in Beirut, Lebanon, are discharged on optimal medical therapy based on the current AHA/ACC guidelines.

**Methods:**

We reviewed the medical records of all patients with ACS who were admitted to the coronary care units (CCU) of two hospitals in Beirut, Lebanon between May and August 2012. Discharge prescriptions were reviewed and rating for the appropriateness of discharge cardiac medications was based on the AHA/ACC guidelines. We assessed whether patients were discharged on antiplatelet therapy, β-blockers, angiotensin converting enzymes inhibitors (ACEIs) or angiotensin receptor blockers (ARBs), statins, and nitrates, unless contraindicated or not tolerated. In addition, we assessed whether patients and/or their caregivers were counseled about their disease(s) and discharge medications.

**Results:**

186 patients with a mean age of 63 ± 11.78 years, 70.4% of which were males, were admitted with ACS and were included in the study. Fifty three (28.5%) patients had ST elevation MI (STEMI), 64 (34.4%) had non-ST-elevation myocardial infarction (NSTEMI) and 69 (37.1%) had unstable angina (USA). Sixty two patients (33.3%) were treated with medical therapy and 124 patients (66.7%) underwent percutaneous coronary intervention (PCI).

Among eligible patients, 98.9% were discharged on aspirin, 89.1% on dual antiplatelet therapy (aspirin + thienopyridine or ticagrelor), 90.5% on a β-blocker, 81.9% on an ACEI or ARB, 89.8% on a statin, and 19.4% on nitroglycerin. Overall, 62.9% of the patients received the optimal cardiovascular drug therapy (the combination of dual antiplatelet therapy, a β-blocker, an ACEIs or an ARB, and a statin), 55.1% were counseled on their disease state(s) and drug therapy, and 92.2% and 55.9% were counseled on smoking cessation and life style changes, respectively.

**Conclusion:**

In patients admitted with ACS, discharge cardiac medications are prescribed at suboptimal rates. Education of healthcare providers and implementation of ACS discharge protocols may help improve compliance with ACC/AHA guidelines. In addition, clinicians should be encouraged to provide adequate patient counseling.

## Background

Coronary artery disease (CAD) is the major leading cause of death worldwide (WHO 2011). According to the World Health Organization, around 17 million people die of coronary diseases each year and over 80% of CAD deaths take place in low and middle-income countries (WHO 2011). Unstable angina (USA), non-ST-elevation myocardial infarction (NSTEMI), and ST-segment elevation myocardial infarction (STEMI) are common manifestations of acute coronary disease and are major causes of hospitalizations (Yang et al. 2006; Setoguchi et al. 2008; Smith et al. 2006). Conversely, the rate has been decreasing during the last 3 decades as a result of better coronary risk factor reduction and better clinical management (Setoguchi et al. 2008; Smith et al. 2006).

The national practice guidelines from the American College of Cardiology (ACC) and American Heart Association (AHA) promote the use of several medical therapies to reduce recurrence of ischemic events and moratlity (Smith et al. 2006; Kushner et al. 2009; Anderson et al. 2013). These medications include dual antiplatelet agents, β-blockers, angiotensin converting enzymes inhibitors (ACEIs) or angiotensin receptor blockers (ARBs), statins, and nitroglycerin (Kushner et al. 2009; Anderson et al. 2013). Death may be prevented during the post-infarction period by different mechanisms in the body; reduction of myocardial ischemia and re-infarction and/or left ventricular dysfunction (LVD) and inhibition of platelet aggregation and rupture (Kushner et al. 2009; Anderson et al. 2013; Frishman and Cheng 1999). To note, β-blockers are beneficial by attenuating the arrhythmogenic potential of damaged myocardium and by reducing myocardial oxygen requirements and thereby the occurrence of ischemia (Frishman and Cheng 1999). While patients recovering from UA/NSTEMI with heart failure, LV dysfunction, hypertension, or diabetes mellitus, should receive an ACEI or an ARB if the former is not tolerated (Class A, Level of evidence: A), the use of these agents is reasonable in the absence of LV dysfunction, hypertension, or diabetes mellitus (Class IIa, Level of Evidence: A) (Anderson et al. 2013). On the other hand, all patients with STEMI should be prescribed at discharge an ACEI (or an ARB for patients who do not tolerate an ACEI) (Kushner et al. 2009). ACEIs and ARBs inhibit the renin -angiotensin system and prevent ventricular remodeling, slow the thickening of the coronary vascular wall, improve subendocardial perfusion as a consequence of lowering left ventricular diastolic pressure, or modulating hormonal factors that influence coronary tone or myocardial perfusion (Kushner et al. 2009; Anderson et al. 2013; Frishman and Cheng 1999). Statins play a role in ACS by involving multiple anti-inflammatory activities to decrease the extent of myocardial necrosis and preserve myocardial viability, ultimately resulting in increased ventricular function (Yamanaka et al. 2012). Yamanaka et al. showed in the Korean Acute Myocardial Infarction Registry (KAMIR) trial that patients with low LDL <100 mg/dl would benefit from statins in reducing the risk of 1-year all-cause death and 1-year major adverse cardiac events (Yamanaka et al. 2012). Antithrombotic therapy is essential to modify the disease process and its progression to death, or recurrent myocardial infarction (MI) (Kushner et al. 2009; Anderson et al. 2013). The use of a combination of dual antiplatelet therapy, β- blockers, ACEIs/ARBs and of statins is essential to all ACS patients with no contraindications to these medications. Nitroglycerin is prescribed at discharge to treat ischemic symptoms and is administered only when needed (Kushner et al. 2009; Anderson et al. 2013).

Despite the general consensus on the efficacy of these drugs for secondary prevention of CAD, adherence to these guidelines is highly variable among physicians (Eagle et al. 2004; Margulis et al. 2011; Lee et al. 2010; Spencer et al. 2001). Previous studies showed that these therapies are neither consistently prescribed when appropriate nor adhered to by patients in the long term (Setoguchi et al. 2008; Eagle et al. 2004; Lappe et al. 2004). In addition, existing research suggest that both filling prescriptions and adherence to cardiac medications are improved by complete hospital discharge recommendations especially if physicians highly encourage their patients to get their medications and provide drug counseling (Eagle et al. 2004; Lappe et al. 2004). Limited studies are available in Lebanon, a third world country, regarding the proper discharge medications and patient education for ACS patients. In our study, we evaluated whether ACS patients were discharged on appropriate cardiac medications (antiplatelets, β-blockers, ACEIs or ARBs, statins, and short-acting nitrates) based on ACC/ AHA guideline (Kushner et al. 2009; Anderson et al. 2013) in 2 major Lebanese hospitals. In addition, we assessed whether patients received education on their disease state, medications, and lifestyle modifications.

## Methodology

This is an observational study that included all ACS patients discharged from the coronary care unit (CCU) from May till August 2012 from two teaching tertiary referral medical centers in Beirut- Lebanon. Data was collected via a review of inpatient medical records and included patient demographics, co-morbidities, in-hospital management, vital signs, laboratory findings, smoking status, and discharge prescriptions. Upon discharge, patients were asked whether they received education about their current disease state, life style modification, and discharge medications. The primary endpoint was to evaluate the use of ACC/AHA guideline recommended cardiac medications (Kushner et al. 2009; Anderson et al. 2013) at hospital discharge in patients admitted for ACS. For the purpose of this study, the prescription of cardiac medications was considered consistent with the guidelines if it was prescribed; and also if it was not prescribed when a contraindication, intolerance, or patient refusal was documented. Secondary endpoints included whether patients received counseling about their disease state, medications, and lifestyle modification.

Continuous variables are expressed as mean ± SD, and categorical variables are presented as frequencies and percentages.

## Results

The study included 186 patients. The baseline characteristics of the study patients are shown in Table 1. The mean age of patients was 63 ± 11.79 years, 70.4% being men. Fifty three (28.5%) of the study patients were diagnosed with STEMI, 64 (34.4%) NSTEMI, and 69 (37.1%) USA. Sixty two patients (33.3%) were treated medically while 124 (66.7%) underwent percutaneous coronary intervention (PCI).

**Table 1:** **Baseline characteristics (n = 186)**

***Demographics***	
**Age**	63 ± 11.79 (years)
**Male**	131 (70.4%)
**Female**	55 (29.6%)
***Diagnosis (%)***	
**STEMI**	28.5
**NSTEMI**	34.4
**USA**	37.1
***Hospital management (%)***	
**Medical treatment**	33.3
**PCI**	66.7
***History of cardiac disease (%)***	
**Hypertension**	93.0
**Atrial fibrillation**	5.9
**Congestive heart failure**	14.0
**Known CAD**	65.6
**Heart valve disease**	1.2
**Previous PCI**	27.4
**Previous CABG**	17.7
***Other comorbidities (%)***	
**Diabetes mellitus**	38.2
**Dyslipidemia**	43.6
**COPD**	6.4
**History of GI ulcer**	5.4
**Previous DVT**	3.2
**CKD**	11.8
**Previous stroke**	5.4
***Life style (%)***	
**Smoking**	51
**Alcohol**	8

CABG: coronary artery bypass graft; GI: gastrointestinal; DVT: deep venous thrombosis.

Some patients had contraindications for receiving certain medications, including bleeding, bradycardia, and hyperkalemia (Table 2). After accounting for contraindications or intolerance, 62.9% of patients were discharged on a combination of dual antiplatelet agents, a β-blocker, an ACEI or ARB, and a statin as recommended by ACC/AHA guideline (Kushner et al. 2009; Anderson et al. 2013). Among eligible patients, 98.9% were appropriately discharged on aspirin, 89.1% on dual antiplatelet therapy (aspirin plus a theinopyridine or ticagrelor), 90.5% on a β-blocker, 81.9% on an ACEI or ARB, and 89.8% on a statin (Table 3).

**Table 2:** **Contraindications for using cardiac medications**

Condition	Contraindicated drug	Number of patients
**Active bleeding**	Antiplatelets	2
**Bradycardia (heart rate ≤ 55 bpm)**	β-blockers	6
**Hyperkalemia**	ACEIs/ARBs	1
**Acute renal injury**	ACEIs/ARBs	3

CPK: creatine phosphokinase.

**Table 3:** Proportion* of patients discharged on cardiac medications

Drug	Aspirin	Thienopyridines or ticagrelor	β-blockers	ACEIs or ARBs	Statins	Nitrates
**Proportion**	182/184	164/184	162/179	149/182	167/186	36/186
**Percentage**	(98.9%)	(89.1%)	(90.5%)	(81.9%)	(89.8%)	(19.4%)

*Proportions describe number of patients discharged on a certain medication divided by the number of patients with no contraindication (eligible) for that medication.

Clopidogrel, ramipril, atorvastatin and bisoprolol were the most commonly prescribed antiplatelet (other than aspirin), ACEI, statin, and β-blocker agents at a rate of 95.7%, 56.4%, 50.9% and 86.4%, respectively. Statins were prescribed at different doses with the moderate and the highest dose being the most commonly prescribed (Table 4).

**Table 4:** **Pattern of statin dosing upon discharge**

High dose statin	Moderate dose statin	Low dose statin
37.7%	37.7%	24.6%

High dose statin: maximal recommended dose of a given statin.

Low dose statin: starting dose of a given statin.

55.1% of the patients were counseled about their disease and drug therapy, 92.2% of smokers about smoking cessation, and 55.9% about life style changes including diet modification and exercise.

## Discussion

The combination of dual antiplatelet agents, a β-blocker, an ACEI or ARB, and a statin is recommended by ACC/AHA guideline for most patients with ACS, unless contraindicated. Our study evaluated the appropriate prescription of cardiac medications upon hospital discharge in patients admitted for ACS and showed that only 62.9% of eligible patients were discharged on the guideline-recommended medications. The prescription rate for aspirin was 98.9%, for dual antiplatelet therapy 89.1%, for β-blockers 90.5%, for ACEIs or ARBs 81.9%, for statins 89.8% and for nitroglycerin 19.35%.

Numerous studies from different countries have reported underuse of optimal medical therapies at hospital discharge. Lee et al. reported in their study done in Korea that the discharge prescription rates of all 4 medications (antiplatelet drugs, β-blockers, ACEIs/ARBs, and statins) was 50.4% (Lee et al. 2010). Wai et al. reported the percentage to be 57% in Australia (Wai et al. 2012). The prescription rate was 48% in China as reported by Bi et al. (Bi et al. 2009). In addition, Al-Zakwani et al. reported in their study done in 6 Middle Eastern countries that 49% of the patients received the quadruple medications at discharge (Al-Zakwani et al. 2011). In France, a study of nationwide registry found that the percentage was only 27% (Danchin et al. 2005). In our study, 62.9% of patients were receiving concomitantly all 4 medications, a percentage that is higher than that described in other countries yet lower than ideal. The higher percentage of prescribing of cardiac medications could be attributed to the fact that we conducted the study in 2 teaching hospitals where compliance with guidelines is expected to be high. Nevertheless, physicians in the 2 hospitals are suboptimally prescribing the cardiac medications as per ACC/AHA guidelines.

The use of each cardiac medication upon hospital discharge in ACS patients varies from one study or country to another. Austin et al. evaluated the use of cardiac medications at hospital discharge for patients with MI in Canada. Overall, 35.6% of patients received an statin, 58.2% ACEIs and 71.0% β-blockers (Austin et al. 2006). Lee et al. reported in their study done in Korea that the discharge prescription rates of antiplatelet drugs, β-blockers, ACEIs/ARBs, and statins were 99.0%, 72.7%, 81.5%, and 77.2%, respectively (Lee et al. 2010). In Australia, Wachtel et al. found that the prescribing rates for ACS medications were: aspirin 90%, β-blockers 55%, ACEIs or ARBs 42%, lipid lowering medication 66% and clopidogrel 64% (Wachtel et al. 2008). Moreover, among ACS patients in Australia, Wai et al. reported that at discharge, 97% received antiplatelet agents, 75% β-blockers, and 78% ACEIs or ARBs (Wai et al. 2012). Furthermore, in Spain, de Velasco et al. reported that at discharge, 94.1% of MI patients received antiplatelet drugs, 59.4% β-blockers, 51.2% ACEIs or ARBs, and 87% received statin therapy (de Velasco et al. 2004). Finally, in New Zealand, Tang et al. reported that upon discharge, the use of aspirin was 98%, β-blockers 80%, ACEIs or ARBs 55%, and statins 70% (Tang et al. 2005). Again, in our study, the percentage of patients discharged on each of the 4 cardiac medications was higher than that described in the above mentioned studies.

In our study, the rate of aspirin prescription is high (98.9%) and comparable, or higher than previous studies and this might be related to the different indications of aspirin in diseases other than ACS. 38.2% of our patients had diabetes mellitus, 5.4% had a history of ischemic stroke and 5.91% had atrial fibrillation which support its high use in these diseases (Association 2011; Samuel Wann et al. 2011). Two patients didn’t receive antiplatelet therapy upon discharge because they had active bleeding during hospital stay which is a major contraindication for its use (Kushner et al. 2009; Anderson et al. 2013). Of the eligible patients, 9.8% received aspirin only and not dual antiplatelet therapy and warfarin or dabigatran were used, along with aspirin, in 4 patients with atrial fibrillation. Some physicians fear the use of triple antithrombotic therapy although studies reported the beneficial combination of the 3 drugs when the anticoagulant has an indication. In a meta-analysis on the use of triple antithrombotic therapy in ACS patients, Gao et al. reported that the triple therapy had significant reduction in ischemic stroke (*P* = 0.0004) as compared with dual antiplatelet therapy but is associated with more bleeding (Gao et al. 2011).

The majority of patients were discharged on clopidogrel (95.7%) while only 6 were discharged on prasugrel (3.7%) and one on ticagrelor (0.6%). This can be explained by the lower cost of clopidogrel and the relatively recent approval of prasugrel and ticagrelor at the time our study was conducted (Jneid et al. 2012).

The rate of prescriptions of β-blockers in our study was 90.5%. Six patients did not receive this therapy due to reported bradycardia (<55 bpm). However, 17 patients didn’t have any contraindication for this therapy and were not discharged on β-blockers. 3.2% of patients were discharged on a calcium channel blocker (CCB) instead of a β-blocker and they were already on a CCB at home. Keeping those patients on CCBs is not justified as it is reasonable to give CCB to patients in whom β-blockers are contraindicated (i.e. bronchospastic disease) for relief of ischemia but not as first line therapy (Kushner et al. 2009; Anderson et al. 2013).

Moreover, 10 patients with chronic obstructive pulmonary disease (COPD) were discharged on β_1_ selective blockers. Chen et al. reported that the use of β-blockers in patients with ACS and COPD was significantly associated with decreased one-year mortality (p < 0.02) given that COPD is moderate and not severe in type (Chen et al. 2001). To note, in our study patients’ COPD symptoms were well controlled and did not worsen with the use of the selective β-blockers.

As compared to other cardiac medications, there was a trend towards lower prescribing rates for ACEIs or ARBs in our study. 81.9% of patients were discharged on ACEIs or ARBs with ramipril being the most commonly prescribed ACEI (56.4%). To note, ramipril, enalapril, captopril and trandolapril are the most studied ACEIs and show comparable efficacy (Frishman and Cheng 1999). TRACE, AIRE, SOLVD and SAVE trials investigate the effect of different ACEIs in ACS patients and they showed reduction in mortality and recurrence of MI (Frishman and Cheng 1999; Kober et al. 1995; AIRE study investigators 1993). A possible explanation to the high use of ramipril is being the formulary ACEI in both hospitals.

In our study, 89.8% of patients were discharged on statins with atorvastatin being the most commonly prescribed (88 out of 167 prescriptions (52.7%)). Despite the relatively high prescription rate of statins in our study, they were not prescribed at the high doses that were proven to offer more protection against major cardiovascular events in clinical trials as compared to lower doses. High dose statin was only prescribed for 37.7% of patients. PROVEIT TIMI-22 and IDEAL trials investigate the role of intensive therapy with pravastatin, atorvastatin and simvastatin as compared to moderate therapy and they show that high doses significantly reduce first occurrence of death, MI, stroke, angina requiring re-hospitalization, or revascularization after ACS event (Murphy et al. 2009; Pedersen et al. 2005).

As for short acting nitrates, only 19.4% of ACS patients were discharged on nitroglycerin. Short acting nitrates are used on as needed basis and are usually not needed after patients undergo successful PCI whereby atherosclerotic coronary arteries get fixed.

Despite some advances in prescribing cardiac drugs upon discharge as per guideline, Eagle et al. reported that discontinuation of therapy was observed at 6-month follow-up in 8% of patients taking aspirin on discharge, 12% of those taking beta-blockers, 20% of those taking ACEIs, and 13% of those taking statins (Eagle et al. 2004). Reasons of non-adherence may be attributed to poor communication and education about the importance of therapy at the time of hospital discharge (Kripalani et al. 2007). In our study, only 55.1% of patients were counseled on their drug therapy which may increase the risk of non-compliance. Hospitals should have a focus discharge counseling on informing patients of major diagnoses, medication changes, dates of follow-up appointments, and provide detailed discussion on the importance, indication and adverse effects of each drug so that patients will adhere to therapy (Albert 2008). In addition, only 55.9% were educated about life style changes and 92.2% of smokers about smoking cessation. Available studies show convincingly the health benefits of lifestyle changes in CAD patients (Iestra et al. 2005).

There are several limitations of our study. First, it was an observational study and done during a relatively short period of time. Second, although we accounted for medication contraindication when assessing guideline adherence, undocumented medication contraindications might have existed. Consequently, we may have underestimated the prescription rates for the drugs studied.

## Conclusion

Hospital discharge offers a major opportunity for quality improvement interventions since it is the linkage point between in-patient and out-patient care. This study provides insights on the pattern of discharge prescriptions of ACS patients in 2 Lebanese hospitals. Despite the beneficial outcomes of cardiac medications, the rate of prescription remains suboptimal and varies among classes where aspirin remains on top of the list followed by β-blockers, statins, and ACEIs or ARBs. Clinicians should provide medical counseling to all ACS patients about their disease and its progression and demand pharmacy counseling for all cardiac medications to ensure patients’ better understanding of the drug indication and importance.
